# Charge to the Graduating Class of the Southern Medical College, March 4th, 1885

**Published:** 1885-04-20

**Authors:** Thomas S. Powell


### CHARGE TO THE GRADUATING CLASS OF THE SOUTHERN MEDICAL COLLEGE MARCH 4TH. 1885, BY PROF. THOMAS S. POWELL, M. D.

My young professional brethren, up to this hour you have been my students, but with the pleasant ceremony of presenting you with a diploma you are now by the laws of Georgia Doctors of Medicine, and your own arbiters of professional status in the future.

As I feel most profoundly the grave and almost sacred significance of a physician's life and duties, I do not think an occasion like this should be made one of levity or frivolity. I would not, however, discourage an ardent enthusiasm in the pursuit of success in your chosen profession, for this is always a most desirable element in any laudable ambition, and in fact, is a divine impetus to great and heroic achievements upon life's fields of honor. But, at the same time, there is a seriousness and dignity about the very nature of the science and practice of medicine that should never be lost sight of in all that pertains to its relations.

Oliver Wendell Holmes says, that he "dare not place any gift, however beautiful, or any success, however brilliant, above the talent or the skill which can relieve a single physical pain, and the self-devotion that lays them at the feet of the humblest fellow creature."

I am sure that you feel the humanity and responsibility of the profession you have chosen, which if regarded in its true and complete aspects, is indeed "a high and holy calling"

I therefore trust that this consciousness of the obligations you now assume, will ever lead you by words and deeds to illustrate the ministry of medicine in its noblest and most exalted significance.

This is best done by a moral observance of all the virtues that exalt and ennoble manhood, as well as by the eminence of the medical skill you may acquire and the faithful discharge of your professional duties towards your fellow men, whether they be of high or low estate. In fact, the noblest exposition of a physician's life is his professional sympathy and benevolence towards the suffering and destitute poor of the community in which he has cast his lot. It is in this direction he can best imitate not only some of the most noted practitioners of medicine in modern times, but can also follow in the footsteps of his Divine Exemplar, whose life was one continuous act of doing good.

It is apparent to all thinking minds that a spirit of irreverence for pure and sacred things, and of lawless indifference to moral obligations, generally, is too prevalent among all classes of people, and is the one great cause of the deplorable demoralization in communities and among individuals of all avocations and professions.

I beseech you, gentlemen, not to let such a spirit enter into your professional career and blight its present fair promise of golden deeds and honorable eminence.

Respect all rights and privileges of your brethren in the profession. Revere the integrity and purity of men's principles, motives and actions; and writing truth and virtue upon your hearts, strive to emulate all the graces of the soul that aid in molding the character of the noble man, the Christian gentleman and physician.

In the distinction of character no man should seek to have a more eminent place than the physician. He may have a medical record of brilliant repute, and attain the highest honors in the profession, but these only form the glittering apex of the fabric, the base of which should be character molded in the solidity of granite, yet with the polish and lustre of Carrara marble. Incorruptible morals should be the corner-stone—firmness, integrity, prudence, truth, tenderness, gentleness, and a sympathetic heart to feel for every ill to which suffering humanity is heir, should be engraved in jewelled characters on the entire structure.

To be a Doctor in the true and original sence of the word you must possess a cultivated heart and mind. Such a man is the noblest creation of Almighty Power, and in the wide universe of things stands indisputably pre-eminent and transcendent. As Mont Blanc, the monarch of mountains, rears proudly and majestically its heaven scaling summit above the clouds and reflects forever the brightest and earliest beams of a gorgeous sun, so do men with highly cultivated hearts and minds shine conspicuous and tower above all of their less favored fellow citizens. Therefore, in the scale of existence, superiority of mind and heart constitutes the only true criterion of greatness, and alone entitles man to the appellation of Doctor of Medicine.

Suffer me then, my young brethren, to entreat you in these. my parting words, to consider the importance of religion to yourselves, your country and the world. Determine to be Christians—always remembering that he only is a real man who discharges his duty in the fear of God. To do this, let me exhort you to resolve this night to live worthy of your noble profession—to do good and avoid vice if you go to the stake. No matter whether right is popular or convenient, adhere to virtue. Make this resolution and then prefer to give your life away before you will budge from it. Let the world, as it will, say you are too rigid—this same world will still respect you. As certainly as God lives in Heaven you will be vindicated and successful.

My young friends, ponder these thoughts, weigh them, and may God sanctify them to great and good results.

Methinks I hear the whisper from your lips: "Vissimus—the day is ours, the work has begun." Oh, that I could explore the mysterious future and promise that it should be to you a delightful stream with fruits and flowers on its banks, that you should see nothing but bright suns, crimson skies and rainbows, verdant hills and luxuriant fields adorned with golden trees and roseate hues. But I can only hope that it will ever have far more of life's sunshine than its clouds. But should you have troubles, be not discouraged. At best, life must have its trials. After all you may do in the cause of truth and the right, even then an unjust punishment may be inflicted upon you.

A great philosopher was once doomed to die. A friend visited him filled with pity.

"I am sorry you are going to die innocent," said the visitor.

"What," returned the philosopher, proudly, "would you have me die guilty?"

What a lesson my young brethren to teach you the great truth that without the Bible and the religion it teaches you can have no solid happiness on earth or hope of felicity hereafter, and that the troubles that beset the good man in most instances prove at last to be the chained lions of good old Bunyan. That God is never asleep, that He loves His children, that He is on their side against all enemies, whether in the shape of flesh or devils; and although He may not slay them as He did the two hundred and fifty princes who rebelled against Moses and Aaron, "with fire from the Lord," or "open the earth and swallow them" as He did Korah, Dathan, and Abiram, still God lives and reigns and He has declared that though hand join in hand, the wicked shall not go unpunished.

Therefore, above all things, fix to-night a high basis for future life and adhere to it. Resolve to go forward from this rostrum, to-night, with a brave heart and manly energy to overcome and conquer every obstacle to your success, and when the result of your action and your energies are seen in your growing prosperity and your upward course, then the demon Jealousy in some dark deceptive bosom, may, perhaps, hurl her darts—point them with destruction—and with the force of slander's tongue, aim them at your fair name; and I regret to say that they will come mainly from members of the medical profession; not from those in the higher and nobler class of Physicians, but in that lower circle, for whom nature and education have done very little in fitting them for their high pretenions. Such men have neither the natural capacity, nor the learning and accomplishments which you and others will have, They seek to run with you in the great race of life, but the clouds of ignorance darken their path, and with jealous rage they fall into the rear, and drag their slow length along.

They see you soar on the wings of fame and fortune, and they too invoke the wings of Genius and the power of talents to bear them up in a little loftier flight. But lo, they have neither the bright pinions of Genius nor the mighty power of Intellect to lift them above the clouds of ignorance, and they grope and trail on, with no higher aim than that of dsepoiling you of the fame and fortune which they can never achieve.

Let me invoke you, my young graduates, to pity an pray for such men, and never condescend to occupy their degraded position by openly contending and discussing with them issues which they will raise, remembering the mousing bat or the hooting owl never attract the notice of the proud eagle, who, heedless of all below him, moves still swiftly on, cleaving the skies in the grandeur of his flight. It is seldom that a falcon towering in her pride of flight is by a mousing owl hawked at and killed.

Let your course be like that of the eagle, all heedless of those below and beneath you; press on to the achievement of a great name and glorious destiny. Do this and you will place yourselves so high in the scale of usefulness and virtue, that the fiery shafts of enemies will never reach your high position.

Finally, gentlemen, you know your duties to yourself, to your profession, to your patients, to your Alma Mater, to each other. Go then, and be eyes to the blind, feet to the lame, and let the blessing of him that is ready to perish come to you. Pursue the course I have suggested. Consecrate your life to honor and usefulness, and whether your life be "robed in sunlight or darkened by trouble and tempest," posterity will embalm your memory, and a greatful world proclaim you a shining light in your own generation, a benefactor to successive generations of mankind. and if I should meet you no more on earth God grant we all "some day" may clasp hands on the fair fields of Eden and teacher and pupils be united forever in one happy. common brotherhood, with God over all.

